# Beef Traceability Between China and Argentina Based on Various Machine Learning Models

**DOI:** 10.3390/molecules30040880

**Published:** 2025-02-14

**Authors:** Xiaomeng Xiang, Chaomin Zhao, Runhe Zhang, Jing Zeng, Liangzi Wang, Shuran Zhang, Diego Cristos, Bing Liu, Siyan Xu, Xionghai Yi

**Affiliations:** 1 Research Institute for Doping Control, Shanghai University of Sport, Shanghai 200438, Chinaxusiyan9715@163.com (S.X.); 2 Technical Center for Animal, Plant and Food Inspection and Quarantine, Shanghai Customs, Shanghai 201210, China; 3 Shanghai Entry and Exit Food and Feed Safety Professional Service Platform, Shanghai 201210, China; 4 Food Technology Institute-Agroindustry Research Center, Hurlingham 1686, Buenos Aires, Argentina; 5 Institute of Science and Technology of Sustainable Food Systems, Hurlingham 1686, Buenos Aires, Argentina

**Keywords:** beef traceability, machine learning, elemental analysis, stable isotope ratio, classification model

## Abstract

Beef, as a nutrient-rich food, is widely favored by consumers. The production region significantly influences the nutritional value and quality of beef. However, current methods for tracing the origin of beef are still under development, necessitating effective approaches to ensure food safety and meet consumer demand for high-quality beef. This study aims to establish a classification model for beef origin prediction by analyzing elemental content and stable isotopes in beef samples from two countries. The concentrations of elements in beef were analyzed using ICP-MS and ICP-OES, while the stable carbon isotope ratio was determined using EA-IRMS. Machine learning algorithms were employed to construct classification prediction models. A total of 83 beef samples were analyzed for the concentrations of 52 elements and the stable carbon isotope ratio. The classification accuracy of the PLS-DA model built on these results was 98.8%, while the prediction accuracy was 94.12% for the convolutional neural network (CNN) and 82.35% for the Random Forest algorithm. The PLS-DA model demonstrated higher classification accuracy compared to CNN and Random Forest, with an explanatory power (R^2^) of 0.924 and predictive ability (Q^2^) of 0.787. Combining the analysis of 52 elements and the stable carbon isotope ratio with machine learning algorithms enables effective tracing and origin prediction of beef from different regions. Key factors influencing beef origin were identified as Fe, Cs, As, δ^13^C, Co, V, Sc, Rb, and Ru.

## 1. Introduction

Beef, as a common type of meat, is widely favored for its unique flavor and rich nutritional value. With increasing economic development, the demand for beef has surged, accompanied by higher consumer expectations for its safety and quality [1]. Argentina is one of the world’s leading beef exporters, predominantly raising Angus cattle through grass-fed practices—both essential attributes of premium-quality beef [2]. In 2023, Argentina exported 683,000 tons of beef globally, with China importing 527,000 tons, accounting for 79% of Argentina’s beef exports, making China its largest beef importer. Due to the high economic value of beef, cases of beef origin fraud are prevalent, causing financial losses and health risks to consumers. For example, some unscrupulous vendors sell beef from low-cost origins as high-priced origin products or adulterate beef with chicken, duck, or pork to increase profit margins [3,4]. Additionally, the illegal use of steroid hormones like trenbolone and clenbuterol during cattle farming to boost yield poses health risks to consumers. For professional athletes, consuming such beef could lead to a positive doping test, potentially jeopardizing their careers [4,5].

Traditional methods for tracing beef origin suffer from low accuracy and limited capability to identify actual production regions, leaving loopholes in food safety [6]. As the beef market continues to expand, it is increasingly crucial to develop efficient origin-tracing methods to ensure the safety of beef production and consumption. Meat traceability primarily relies on isotope and elemental analysis techniques. Environmental factors such as soil, water, and climate leave unique elemental and isotopic “fingerprints” in the muscle tissue of cattle. By analyzing these characteristics, the geographical origin of beef can be inferred. Stable isotope and elemental analyses are commonly used, with EA-IRMS (Elemental Analysis–Isotope Ratio Mass Spectrometry) and ICP-MS/OES (Inductively Coupled Plasma Mass Spectrometry/Optical Emission Spectrometry) being the most prevalent tools [7]. EA-IRMS measures isotope ratios of elements like carbon, nitrogen, hydrogen, and oxygen by combusting samples into their elemental components [8]. The carbon isotope ratio (δ^13^C) reflects the photosynthetic pathways of plants consumed by the cattle, providing insights into their feed sources [9]. Carbon isotopic characteristics of beef can distinguish between grass-fed and grain-fed beef and identify the cattle’s rearing environment. Argentina’s cattle are predominantly grass-fed, relying on pastures (C3 plants), which use the Calvin cycle for photosynthesis [10]. The carbon isotope ratio of C3 plants typically ranges from −22‰ to −30‰, reflecting a preference for the lighter carbon isotope (^12^C) over the heavier isotope (^13^C). In contrast, Chinese cattle are mainly grain-fed, with feed derived from corn, sorghum, and other C4 plants that employ the Hatch–Slack pathway for photosynthesis. C4 plants exhibit δ^13^C values of −10‰ to −14‰, and these differences in feed ultimately result in distinct isotopic fingerprints in the beef, providing a natural traceability tool [11,12,13].

In recent years, machine learning, a cornerstone of artificial intelligence, has emerged as a powerful tool for data processing, analysis, and prediction in food science [14,15]. PLS-DA (Partial Least Squares–Discriminant Analysis) is a multivariate statistical method widely applied in food traceability for its ability to extract relevant features from high-dimensional data and classify the origin and variety of food accurately [16]. It is particularly suitable for handling complex chemical data, such as spectral analysis and isotope ratios, and has been extensively used in the traceability of wine, tea, and olive oil [17]. For instance, in 2021, Xu et al. analyzed the carbon, nitrogen, and oxygen isotope ratios, 51 elements, and 35 fatty acids in milk powder samples from three regions, constructing a stable multivariate model (R^2^X = 0.693, Q^2^ = 0.854) for origin identification [18]. In 2022, Dehelean et al. measured the carbon, hydrogen, and oxygen isotope ratios and 28 elements in Romanian pork and chicken samples, using PLS-DA to establish traceability models with accuracies of 71.8% and 93.8%, respectively, and feeding style identification models with accuracies of 100% and 98% [19]. Convolutional neural networks (CNNs), a deep learning model, are increasingly used in food traceability due to their ability to efficiently extract local and global features through convolution and pooling operations [20]. In one study, Weng et al. utilized CNNs alongside support vector machines, Random Forests, and K-nearest neighbors (KNN) to analyze hyperspectral imaging data of lamb, successfully constructing geographical origin and breed identification models [21]. Random Forests, a decision tree-based ensemble learning method, are another popular machine learning approach in food science [22]. For example, Elisabete A. et al. analyzed elemental data from beef samples from five Brazilian regions, using Random Forests, multilayer perceptrons, and regression trees to classify beef origins, confirming significant regional differences in Brazilian beef [23].

This study addresses the critical need for robust food traceability systems by developing an innovative origin verification approach for beef products. Integrating multi-regional sampling from China and Argentina with advanced analytical techniques (ICP-MS, ICP-OES for 52 elemental profiles, and EA-IRMS for carbon stable isotope ratios), we established a comprehensive analytical framework that significantly enhances geographical discrimination accuracy. The creation of a machine learning-powered classification model not only demonstrates high-precision origin identification between these major beef-producing nations but also establishes a suitable methodology combining multi-elemental fingerprinting with stable isotope analysis. This breakthrough provides both technical support for combating food fraud and a scientific foundation for global meat traceability protocols while advancing the application of chemometric approaches in food authentication. Subsequent research will focus on model optimization for wider geographical coverage and the integration of complementary biomarkers to strengthen verification reliability.

## 2. Results

### 2.1. Analysis of Elemental Data

Using ICP-MS and ICP-OES, we determined the concentrations of 52 elements in 83 beef samples. The results are summarized in Table 1.

Among the 52 elements, 23 elements showed significant differences (*p* < 0.005), including Mg, P, Sc, K, V, Fe, Co, As, Rb, Ru, Rh, Cs, La, Ce, Nd, Sm, Eu, Gd, Tb, Dy, Yb, Tl, and Pb. The *p*-values for K, Fe, Rb, and Cs were less than 0.0001.

The observed differences in magnesium (Mg) content may be attributed to the prevalence of hypomagnesemia in cattle from central Argentina, which results in lower magnesium levels in Argentine beef compared to Chinese beef [24]. Significant differences in elements such as Fe, Cs, and Co have also been confirmed through molecular activation analysis and machine learning [25]. Additionally, previous studies have indicated that elements such as Dy, Fe, Rb, Tl, V, and 14 other elements are important factors for identifying the geographical origin of beef in traceability studies across different countries [26]. Combining findings from other studies with our experimental results, it can be concluded that there are significant differences in the elemental composition of beef from China and Argentina.

### 2.2. Analysis of Carbon Stable Isotope Ratios

**Figure 1:**
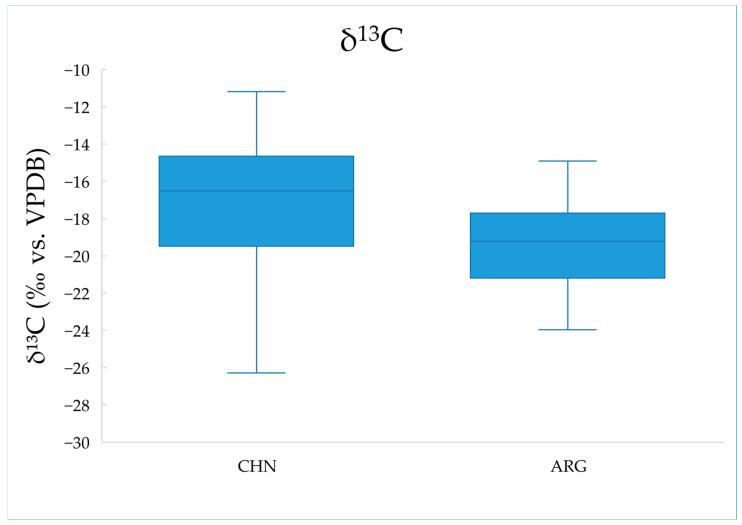
Boxplot of δ^13^C for beef samples from China and Argentina.

We measured the carbon stable isotope ratios of 83 samples. The mean δ^13^C values and standard deviations of samples from China and Argentina are summarized in Table 2. A boxplot illustrating the δ^13^C distribution is shown in Figure 1.

**Figure 2:**
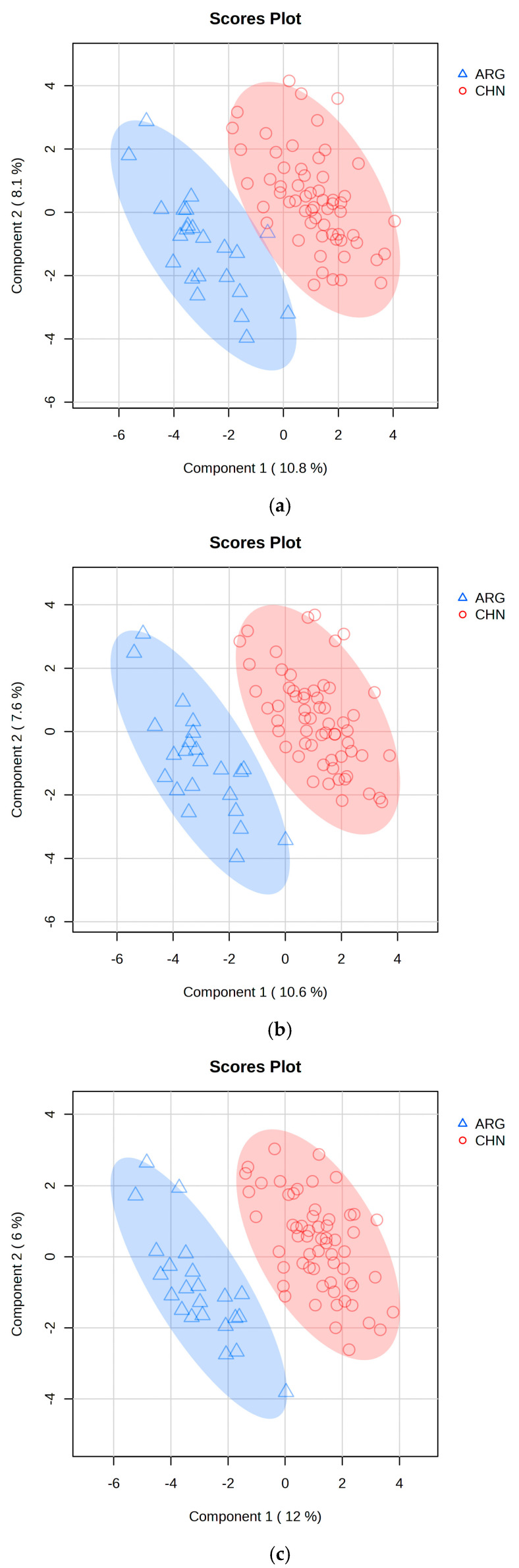
PLS-DA classification model of Chinese and Argentine beef samples. (**a**) is the PLS-DA classification plot based on elemental properties; (**b**) is the PLS-DA classification plot based on both elemental and carbon stable isotope properties; (**c**) is the PLS-DA classification plot after removing redundant variables from (**b**). The blue and red shadow is the 95% confidence interval of PLS-DA models, it means the model’s classification of beef samples origin.

Through the measurement of samples, we found that the δ^13^C values of beef samples from China range from −27.23‰ to −11.18‰, while those from Argentina range from −23.97‰ to −14.91‰, with average values of −17.52‰ and −19.58‰, respectively. The standard deviation of the carbon stable isotope ratio in Argentine beef samples was 2.42, whereas the standard deviation of Chinese beef samples was larger, at 3.81. The boxplot (Figure 2) shows that the δ^13^C values of Argentine beef are lower and more concentrated, while those of Chinese beef are higher and more dispersed. The standard deviation of Chinese beef is larger compared to Argentine beef, and the range between the maximum and minimum values is also greater, confirming the more uniform farming practices of Argentine cattle compared to China. In Argentina, most cattle are fed with C3 plants during the rearing process, resulting in a lower average δ^13^C value in Argentine beef compared to Chinese beef. In contrast, cattle farming methods in China are more diverse in terms of geography and feeding practices. China has both grass-fed cattle primarily fed on C3 plants and grain-fed cattle that are often provided with C4 plants as feed. As a result, the average δ^13^C value of Chinese beef is higher than that of Argentine beef [27,28,29]. There is a significant difference in δ^13^C values between Chinese and Argentine beef, suggesting that δ^13^C could serve as a potential indicator for distinguishing beef origins among the existing analysis variables.

### 2.3. PLS-DA Classification Model

The classification model is shown in Figure 2a. We constructed the PLS-DA classification model using the elemental content data of beef samples. In the model, the samples are clearly divided into two clusters, with a 95% confidence interval serving as the threshold to differentiate the beef samples from the two regions. K-fold cross-validation is an effective method for evaluating PLS-DA models. In k-fold cross-validation, the original dataset or training dataset is divided into k folds (subsets), and the model is trained k times (iterations), resulting in k performance estimates. In each iteration, one fold (validation set) is used to evaluate the model, and the remaining k-1 folds are used for training. In this study, due to the sample size being less than 100, 10-fold cross-validation was chosen to obtain the best cross-validation results [30,31]. Performance evaluation depends on the accuracy, R^2^ (coefficient of determination), and Q^2^ (predictive R^2^) obtained from the 10 validation iterations. These represent the proportion of correctly classified samples out of the total number of samples, the degree of fit between the model and the training data, and the model’s ability to predict unseen data. Among these, Q^2^ reflects the model’s predictive performance in cross-validation. A high Q^2^ value indicates that the model not only fits well on the training set but also has good predictive capability on new data [32]. When using the 52 elemental content features as variables, the classification accuracy for the beef from the two regions was 97.8%, with an R^2^ of 0.915 and a Q^2^ of 0.706.

The PLS-DA model can also optimize classification results by removing redundant variables. The classification ability of the PLS-DA model depends on the correlation between the variables and the class labels. If there are many irrelevant or redundant variables, they may be treated as noise and introduced into the model, which could interfere with the model, reducing its performance and robustness [33,34,35]. VIP (Variable Importance in Projection) is an indicator used to assess the importance of each variable (feature) to the model. It helps identify which variables contribute most to the model’s predictive ability, allowing the selection of variables that have the most impact on classification or regression results. Variables with low VIP values are less important to the model and can be discarded to improve classification accuracy [36,37]. By evaluating the VIP values of the model and re-modeling, redundant variables such as Tm, Au, Pr, Sb, Y, Tb, Eu, and Sm, which contribute less to the model, were removed. The classification model results after this adjustment are shown in Figure 2c. From the results, it can be seen that the samples within the same region are now more tightly clustered, the 95% confidence interval has narrowed, and the differentiation between regions is more prominent. The 10-fold cross-validation results are shown in Table 2, where the classification accuracy for beef from the two regions is 98.8%, with an R^2^ of 0.925 and a Q^2^ of 0.787. Compared to the model trained with single-variable data, R^2^ and Q^2^ have improved by 1.05% and 11.47%, respectively. This demonstrates that removing redundant variables significantly enhances the model’s predictive capability.

**Figure 3:**
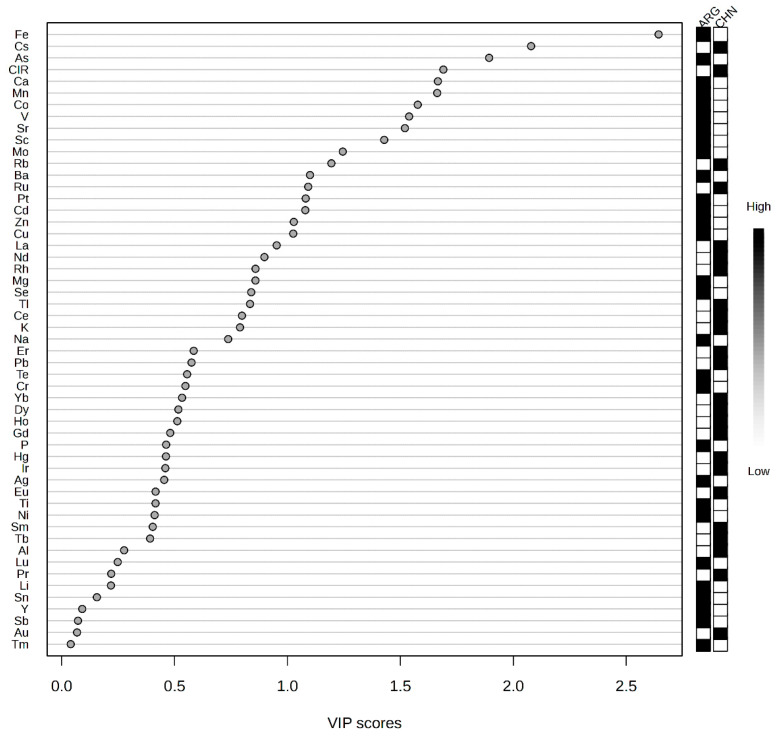
VIP score plot in the PLS-DA model.

In addition, in the PLS-DA model, variables with higher VIP values contribute more to the model. Therefore, VIP values can serve as an important criterion for selecting key influencing factors of beef origin [38,39]. Figure 3 shows the ranking of VIP scores in this PLS-DA model. In this study, variables with VIP scores greater than 1.0, such as Fe, Cs, As, δ^13^C, Ca, Mn, Co, V, Sr, Sc, Mo, Rb, Ba, Ru, Pt, Cd, Zn, and Cu, significantly contribute to the model. These variables can be considered key influencing factors for beef origin. Among them, Fe is the most distinct variable. Fe, Cs, As, Co, V, Sc, Rb, and Ru are also the elements with the largest differences in the element data *t*-test results (*p* > 0.05). However, not every element with a large *t*-test result is a key influencing factor. This is because VIP values take into account not only the significance of variables but also their explanatory power within the model. If a variable contributes little to the overall variation in the model, and factors such as small sample size or data imbalance cause certain variables to appear significant in *t*-tests but not adequately represent their importance in multivariate model analysis, even if they are significant in individual *t*-tests, their VIP values might still be low. For instance, Rh is one such example. δ^13^C ranks fourth in the VIP value list, indicating that carbon stable isotope ratios are a significant contributor to this classification model and are a crucial factor in distinguishing beef from different regions.

### 2.4. CNN Classification Model

The convolutional neural network (CNN) classification model was constructed using the TensorFlow library with Python 3.12.6, with the sample data randomly split into training and test sets in an 80:20 ratio. CNNs achieve efficient feature learning and classification prediction by extracting local features from the input data and progressively abstracting them through multiple layers. The basic structure of the model consists of the input layer, convolutional layers, pooling layers, fully connected layers, activation functions, and the output layer. The input layer receives the raw data, the convolutional layers use filters (kernels) to extract local features, the pooling layers reduce the dimensionality of the feature maps, the fully connected layers map the extracted features to classification results, the ReLU activation function introduces non-linearity, and the output layer produces the classification probability or class labels based on the specific task. Based on the dataset characteristics, two convolutional layers were used to extract features, two pooling layers were employed to reduce dimensions, two Dropout layers were added to prevent overfitting, and two fully connected layers were utilized to complete the classification prediction. Finally, the Softmax activation function was applied to convert the output into a probability distribution over the classes, thus completing the CNN classification model [40,41,42].

**Figure 4:**
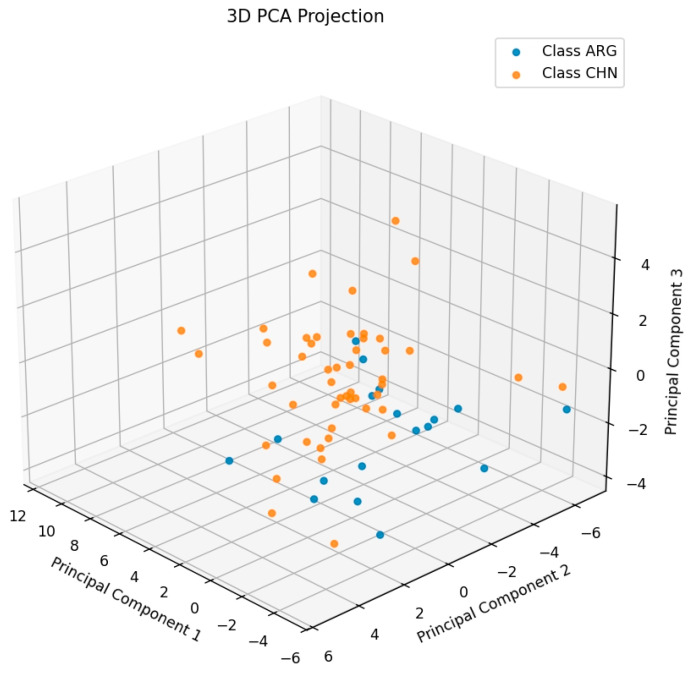
CNN model classification plot.

The results of the classification model trained on the training set are visualized in Figure 4. As can be seen, after feature extraction, the dataset shows a clear trend of classification. While the CNN model’s predictive ability cannot be fully visualized in a 3D plot, PCA can still be used to preliminarily assess the CNN’s ability to extract features. The prediction results for the test set are shown in Table 3. It is evident that the CNN performs excellently in classifying the test samples. Out of 17 samples, only 1 was classified incorrectly, yielding an overall accuracy of 94.12%. Moreover, the confidence for most correctly predicted samples was over 90%.

### 2.5. Random Forest Classification Model

The classification prediction results for the test set are shown in Table 4. In this study, 100 decision trees were used to construct the Random Forest classification model. Typically, the more trees in the model, the more stable its performance, though the computational time also increases. The training classification accuracy of this model was 100%, and the validation set accuracy was 78.57%. The model’s classification prediction ability is weaker than that of the CNN model. For Argentine beef classification, the accuracy was 100%, while for Chinese beef samples, the accuracy was 40%, resulting in an overall prediction accuracy of 82.35%.

**Figure 5:**
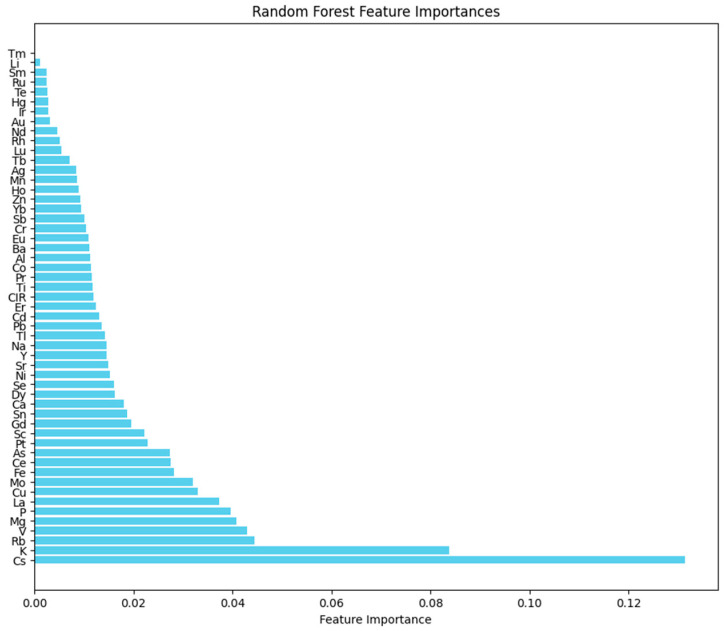
Feature importance in the Random Forest classification model.

In the Random Forest algorithm, feature importance is used to assess the contribution of each sample attribute to the classification model (Figure 5) [43,44]. The top ten ranked variables in terms of importance are Cs, K, Rb, V, Mg, P, La, Cu, Mo, and Fe. Among these, K, Cs, Rb, V, Mg, P, La, and Fe overlap with the VIP values from the PLS-DA model and the elements with the most significant differences in the elemental data. Therefore, these eight variables are key factors that differentiate beef origin. The Random Forest Feature Importances are shown in Figure 5, where the contribution of sample attributes to the model is ranked from smallest to largest. The top ten variables in terms of importance are Cs, K, Rb, V, Mg, P, La, Cu, Mo, and Fe. The classification prediction results for the test set are presented in Table 5, with an overall prediction accuracy of 82.35%.

**Figure 6:**
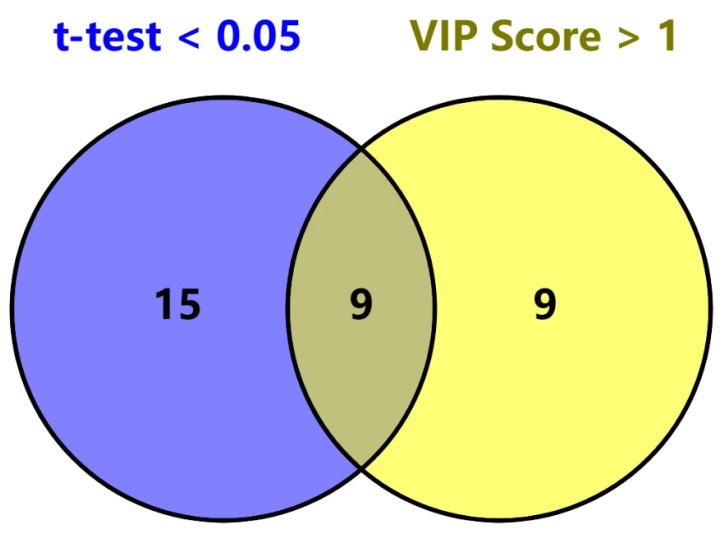
Venn diagram for selecting key influencing factors in beef traceability.

Overall, significant differences exist between Chinese and Argentine beef in terms of elemental content and carbon isotope levels, and these differences can be utilized to construct classification models through machine learning, enabling accurate beef classification. Among all the models used in this study, the PLS-DA model constructed using MetaboAnalyst achieved the highest classification accuracy (97.5%), followed by the CNN model (94.12%) and the Random Forest model (82.35%). By selecting variables with VIP values greater than 1 from the PLS-DA model, as well as variables with significant differences in elemental and carbon stable isotope analysis results (*p* < 0.05), a Venn diagram was constructed. The results are shown in Figure 6. This method identified nine key variables: Fe, Cs, As, δ^13^C, Co, V, Sc, Rb, and Ru. These variables show significant differences between the beef samples from the two regions and contribute significantly to the origin traceability model. Therefore, these nine variables can be regarded as key influencing factors for beef origin traceability.

## 3. Material and Methods

### 3.1. Materials and Reagents

HPLC-grade methanol was purchased from TEDIA (Fairfield, OH, USA). Chloroform (analytical grade) was obtained from Sinopharm Chemical Reagent Co., Ltd. (Beijing, China). IAEA-600 caffeine (δ^13^C = −27.771‰) was supplied by the International Atomic Energy Agency (Vienna, Austria). Standard solutions of Ir, Pt, Au, Ag, V, Co, Te, Rb, Ru, Cs, and As (1000 µg/mL) and four internal standards (Ge, Rh, In, and Bi) were purchased from o2si (South Carolina, NC, USA). Standard solutions for other elements (1000 µg/mL) and nitric acid were procured from the National Nonferrous Metals and Electronic Materials Analysis and Testing Center (Beijing, China).

### 3.2. Methods

#### 3.2.1. Sample Collection

Beef samples were collected from local markets in various regions of Argentina and provinces of China. A total of 83 beef samples were collected, including 60 samples from different provinces in China (Heilongjiang, Jilin, Xinjiang, Gansu, Inner Mongolia, Henan, Yunnan, Ningxia, Qinghai, Jiangsu, Sichuan, and Shandong) and 23 samples from different provinces in Argentina (Buenos Aires, Santa Fe, La Pampa, and Entre Ríos). After the sample collection, they were stored in a −20 °C freezer, and the subsequent analysis was completed within 30 days.

#### 3.2.2. Preparation for Elemental Determination

To determine the elemental content of beef samples, appropriate preprocessing was conducted to meet ICP-MS/OES requirements. Using microwave digestion, 1.0 g of each beef sample (accurate to 0.001 g) was weighed and treated according to the national standard GB 5009.268-2016 [45]. Five milliliters of nitric acid were added to the weighed samples, followed by sealing and vortexing for 30 s. The samples were then placed in a preheated microwave digestion system. The digestion program was set as follows: initial temperature of 120 °C held for 5 min, increased to 150 °C for 10 min, and then raised to 190 °C for 20 min. After digestion, the mixture was cooled to room temperature, filtered using a funnel to remove solid residues, and diluted to 50 mL with deionized water for analysis.

#### 3.2.3. Preparation for Carbon Stable Isotope Ratio Determination

Since isotope fractionation occurs during fat synthesis, and fat content varies across beef samples, defatting was necessary to eliminate the influence of fat on carbon isotope ratio determination. For this, 300 g of beef sample was minced, and 15.0 g of each sample was evenly spread in a petri dish. The samples were freeze-dried at −45 °C using a benchtop freeze-dryer and then ground into powder. Following the rapid sample preparation method for beef isotope determination by Zhao et al. [46], 5 mL of a chloroform–methanol solution (2:1, *v*:*v*) was added to the powdered sample in a centrifuge tube. The mixture was vortexed for 10 min (2500 rpm) and centrifuged at 4 °C using an Eppendorf 5810R centrifuge. The fat-containing supernatant was discarded, and the solid residue was collected. This defatting process was repeated twice. Finally, the samples were dried overnight in an oven at 50 °C. Using a microanalytical balance (XPE26C, Mettler Toledo, Switzerland), 80–130 µg of defatted, dried sample powder was weighed into tin capsules (Sn, 8 mm × 5 mm, Thermo Fisher, Waltham, MA, USA), sealed, and formed into a ball for carbon isotope ratio determination. Each sample was measured six times, and the mean value was used for accurate δ^13^C determination.

#### 3.2.4. Elemental Measurements by ICP-OES and ICP-MS

The elemental content of beef samples was measured using an Agilent 5100 ICP-OES (Agilent Technologies, Santa Clara, CA, USA) for Na, K, Mn, P, Zn, Ca, Fe, and Mg, while 44 other elements were analyzed using an Agilent 7900 ICP-MS (Agilent Technologies, USA). Each sample was analyzed in duplicate for all element measurements.

Parameters for ICP-OES included the following: replicate time of 3, pump speed of 12 rpm, uptake delay of 15 s, reading time of 3 s, RF power of 1.2 kW, stabilization time of 10 s, and observation modes (radial and axial simultaneous view). The plasma gas flow rate was set to 12.0 L/min, the nebulizer gas flow rate to 0.70 L/min, the auxiliary gas flow rate to 1.00 L/min, and the additional air flow rate to 0.00 L/min. The detection range was 0.01–0.7 mg/100 g and 0.03–3 mg/100 g.

Parameters for ICP-MS included the following: peak hopping scan mode, 3 points per peak, reading time of 3 s, sample depth of 10 mm, spray chamber temperature of 2 °C, sample uptake speed of 0.5 r/s, carrier gas and hydrogen gas flow rates of 0.82 mL/min and 4 mL/min, dilution plasma flow rate of 0.3 L/min, and RF power of 1600 W. The detection and calibration ranges were 0.03 µg/kg to 0.5 mg/kg and 0.1 µg/kg to 2 mg/kg, respectively.

#### 3.2.5. δ^13^C Measurements by EA-IRMS

Carbon stable isotope ratios (δ^13^C) of beef samples were measured using EA-IRMS (Delta V Advantage, Thermo Scientific, Waltham, MA, USA), with each sample analyzed six times. The obtained δ^13^C values were normalized to the international standard Vienna Pee Dee Belemnite (V-PDB) using laboratory working standards calibrated against V-PDB. Parameters for the elemental analyzer were as follows: oxidation furnace temperature of 980 °C, column temperature of 50 °C, helium carrier gas flow rate of 100 mL/min, oxygen flow rate of 175 mL/min, helium purge gas flow rate of 180 mL/min, and oxygen injection time of 4 s. IRMS conditions included the following: electron ionization mode (EI), ion source voltage of 3.07 kV, vacuum of 1.6 × 10⁻⁶ mbar, and gas pressures of 4 bar (helium, oxygen, carbon dioxide) and 8 bar (air). The δ^13^C values were expressed in per mil (‰) as follows: δ = [(Rsample − Rstandard)/Rstandard] × 1000, where R represents the ^13^C/^12^C ratio of the sample or standard. To ensure accuracy and stability, IAEA-600 (caffeine, δ^13^C = −27.771‰) was used to calibrate the instrument. The average deviation between measured and standard values was less than 0.3‰, and the standard deviation of parallel measurements was below 0.3‰.

#### 3.2.6. Statistical Analysis

The data were statistically analyzed using Microsoft Excel, with the *t*-test performed using Welch’s *t*-test, and the two-tailed *p*-value was calculated. PLS-DA combines the advantages of Principal Component Analysis (PCA) and Partial Least Squares (PLS) regression and is a supervised learning method for handling multivariate data. It is suitable for classification tasks when there are significant correlations and collinearity between samples. We constructed the PLS-DA classification model using MetaboAnalyst 6.0. In the initial data preprocessing, we used log transformation and mean centering to normalize the raw data, making it easier for further analysis. The convolutional neural network (CNN) classification model was constructed using Python 3.12.6 and TensorFlow library, with the sample data randomly split into training and test sets in an 80:20 ratio. CNN achieves efficient feature learning and classification prediction by extracting local features from the input data and progressively abstracting them through multiple layers. The basic structure of the model consists of the input layer, convolutional layers, pooling layers, fully connected layers, activation functions, and the output layer. The input layer receives the raw data, the convolutional layers use filters (kernels) to extract local features, the pooling layers reduce the dimensionality of the feature maps, the fully connected layers map the extracted features to classification results, the ReLU activation function introduces non-linearity, and the output layer produces the classification probability or class labels based on the specific task. Based on the dataset characteristics, two convolutional layers were used to extract features, two pooling layers were employed to reduce dimensions, two Dropout layers were added to prevent overfitting, and two fully connected layers were utilized to complete the classification prediction. Finally, the Softmax activation function was applied to convert the output into a probability distribution over the classes, thus completing the CNN classification model. The Random Forest classification model was constructed using Python 3.12.6 and the Scikit-learn library, with the sample data randomly split into training and external test sets in an 80:20 ratio. Random Forest is based on the decision tree concept, where multiple decision trees are built, and their outputs are integrated to perform classification or regression analysis. The core idea is to combine several weak classifiers (decision trees) into a strong classifier, which improves the model’s accuracy and robustness.

## 4. Conclusions

This study evaluated the elemental and carbon stable isotope characteristics of 83 beef samples (60 from different regions in China and 23 from Argentina). Based on multi-element and stable isotope ratios, three classification prediction models—PLS-DA, convolutional neural network (CNN), and Random Forest—were established. The results showed that these three models were effective in distinguishing the origin of beef from China and Argentina. Among them, the PLS-DA model achieved the highest classification accuracy of 97.5%, with an R^2^ of 0.924 and a Q^2^ of 0.743, indicating strong model performance. The model analysis identified nine key influencing factors—Fe, Cs, As, δ^13^C, Co, V, Sc, Rb, and Ru—that had the greatest impact on beef origin traceability, providing valuable data support for further research on beef product traceability.

## Figures and Tables

**Table 1:** Comparison of the concentrations of 52 elements in beef samples from different origins.

Elements	Unit	CHN (n = 60)	ARG (n = 23)	CHN Max	CHN Min	ARG Max	ARG Min	*t*-Test Result
		Concentration						
Li	mg/kg	0.796 ± 2.707	0.486 ± 0.870	16.533	<0.000	2.778	<0.000	0.4328
Na	mg/100 g	58.421 ± 44.472	50.555 ± 15.529	322.23	21.51	78.68	27.59	0.2362
Mg *	mg/100 g	18.293 ± 3.059	15.486 ± 3.563	23.31	7.48	21.37	8.32	0.002
Al	mg/kg	13.231 ± 32.063	5.630 ± 5.230	195.049	<0.000	21.492	<0.000	0.0803
P *	mg/100 g	156.554 ± 26.276	128.242 ± 30.410	209.93	62.84	169.33	64.08	0.0004
K *	mg/100 g	301.091 ± 53.428	229.876 ± 53.821	367.87	121.97	317.51	124.53	<0.0000
Ca	mg/100 g	9.966 ± 5.063	10.850 ± 3.152	40.29	2.95	18.45	6.65	0.344
Sc *	mg/kg	0.165 ± 0.084	0.227 ± 0.124	0.496	0.016	0.446	0.019	0.0326
Ti	mg/kg	4.856 ± 2.333	4.267 ± 2.183	12.352	<0.000	12.298	0.663	0.2866
V *	μg/kg	0.036 ± 0.020	0.105 ± 0.136	0.101	<0.000	0.431	0.003	0.023
Cr	mg/kg	0.476 ± 0.433	0.435 ± 0.222	2.635	0.106	1.189	<0.000	0.5739
Mn	mg/100 g	17.211 ± 5.139	18.787 ± 5.269	30.53	5.84	31.26	8.73	0.2268
Fe *	mg/100 g	1.979 ± 0.619	2.654 ± 0.721	3.46	0.69	4.69	1.01	0.0003
Co *	μg/kg	0.049 ± 0.028	0.065 ± 0.028	0.135	<0.000	0.124	0.022	0.0292
Ni	mg/kg	0.125 ± 0.153	0.161 ± 0.337	0.618	<0.000	1.671	<0.000	0.6234
Cu	mg/kg	11.769 ± 3.182	12.870 ± 7.082	19.288	3.884	26.763	<0.000	0.479
Zn	mg/100 g	4.214 ± 1.321	4.029 ± 1.424	7.05	1.12	7.8	1.33	0.5935
As *	μg/kg	0.209 ± 0.094	0.370 ± 0.272	0.558	0.071	1.124	<0.000	0.0469
Se	mg/kg	2.362 ± 1.532	2.560 ± 1.394	9.421	0.607	6.734	<0.000	0.5763
Rb *	mg/kg	8.053 ± 4.785	4.092 ± 2.510	22.382	1.903	9.652	1.422	<0.0000
Sr	mg/kg	6.551 ± 2.804	7.477 ± 2.668	13.965	0.776	14.086	4.457	0.1706
Y	μg/kg	0.058 ± 0.055	0.042 ± 0.030	0.231	<0.000	0.125	<0.000	0.094
Mo	mg/kg	0.075 ± 0.056	0.119 ± 0.103	0.281	<0.000	0.337	0.008	0.064
Ru *	μg/kg	0.014 ± 0.034	0.000 ± 0.000 ^a^	0.17	<0.000	0.001	<0.000	0.003
Rh *	μg/kg	0.002 ± 0.003	0.001 ± 0.001	0.012	<0.000	0.005	<0.000	0.0024
Ag	μg/kg	1.354 ± 1.233	1.262 ± 1.017	6.615	0.245	4.96	0.267	0.7289
Cd	μg/kg	0.017 ± 0.012	0.022 ± 0.017	0.05	<0.000	0.082	<0.000	0.1673
Sn	μg/kg	0.045 ± 0.030	0.039 ± 0.033	0.126	<0.000	0.163	<0.000	0.452188
Sb	μg/kg	0.048 ± 0.112	0.026 ± 0.012	0.848	<0.000	0.053	0.01	0.1524
Te	μg/kg	0.003 ± 0.012	0.003 ± 0.010	0.07	<0.000	0.037	<0.000	0.998
Cs *	mg/kg	0.539 ± 0.562	0.097 ± 0.082	2.835	0.062	0.287	0.021	<0.0000
Ba	mg/kg	2.087 ± 1.309	2.099 ± 0.665	10.467	0.124	3.734	1.298	0.9537
La *	mg/kg	0.321 ± 0.722	0.095 ± 0.168	4.729	0.003	0.682	<0.000	0.0262
Ce *	μg/kg	0.126 ± 0.140	0.066 ± 0.053	0.831	0.001	0.22	<0.000	0.0056
Pr	μg/kg	0.014 ± 0.014	0.011 ± 0.010	0.073	<0.000	0.035	<0.000	0.2524
Nd *	μg/kg	0.060 ± 0.048	0.034 ± 0.034	0.2	<0.000	0.132	<0.000	0.0074
Sm *	μg/kg	0.015 ± 0.019	0.008 ± 0.010	0.105	<0.000	0.042	<0.000	0.0469
Eu *	μg/kg	0.023 ± 0.027	0.014 ± 0.010	0.18	0.004	0.042	0.002	0.0288
Gd *	μg/kg	0.017 ± 0.022	0.009 ± 0.013	0.107	<0.000	0.049	<0.000	0.0491
Tb *	μg/kg	0.005 ± 0.005	0.003 ± 0.003	0.017	<0.000	0.011	<0.000	0.0128
Dy *	μg/kg	0.014 ± 0.012	0.009 ± 0.009	0.049	<0.000	0.028	<0.000	0.0231
Ho	μg/kg	0.003 ± 0.003	0.002 ± 0.003	0.015	<0.000	0.012	<0.000	0.1663
Er	μg/kg	0.011 ± 0.008	0.007 ± 0.007	0.034	<0.000	0.024	<0.000	0.0586
Tm	μg/kg	0.001 ± 0.002	0.001 ± 0.001	0.01	<0.000	0.003	<0.000	0.1684
Yb *	μg/kg	0.007 ± 0.007	0.004 ± 0.005	0.037	<0.000	0.013	<0.000	0.0464
Lu	μg/kg	0.001 ± 0.001	0.001 ± 0.001	0.005	<0.000	0.005	<0.000	0.9613
Ir	μg/kg	0.016 ± 0.021	0.008 ± 0.015	0.109	<0.000	0.036	<0.000	0.061
Pt	μg/kg	0.047 ± 0.054	0.069 ± 0.066	0.262	<0.000	0.238	<0.000	0.1587
Au	μg/kg	0.035 ± 0.056	0.019 ± 0.038	0.182	<0.000	0.097	<0.000	0.1401
Tl *	μg/kg	0.007 ± 0.020	0.001 ± 0.001	0.125	<0.000	0.004	<0.000	0.0469
Hg	μg/kg	0.002 ± 0.008	0.000 ± 0.002 ^b^	0.038	<0.000	0.011	<0.000	0.0761
Pb *	mg/kg	0.448 ± 0.477	0.256 ± 0.177	2.559	0.066	0.844	<0.000	0.009

Note: Elements marked with an asterisk (*) indicate a *t*-test result of *p* < 0.05, representing a statistically significant difference. ^a^ Additionally, due to the low Ru content in the Argentine samples, the average value in Table 1 is shown as 0.000, while the actual average Ru content is 4.35 × 10⁻^5^ μg/kg. ^b^ Similarly, the actual average Hg content in the Argentine samples is 4.78 × 10⁻^4^ μg/kg.

**Table 2:** Differences in carbon stable isotope ratios (δ^13^C) between beef samples from China and Argentina.

Location	δ^13^C AVG	δ^13^C STD	Max	Min	*t*-Test
China (n = 60)	−17.52	3.81	−11.18	−27.23	0.005
Argentina (n = 23)	−19.58	2.42	−14.91	−23.97	

**Table 3:** Ten-fold cross-validation results of the PLS-DA model.

Data Source	Accuracy	R^2^	Q^2^
Elements	97.8%	0.915	0.706
Elements and δ^13^C	97.5%	0.924	0.743
Elements and δ^13^C with redundant variables removed	98.8%	0.925	0.787

**Table 4:** Prediction results of the CNN testing set.

Samples Number	Origin Class	Predicted Class	Confidence	Accuracy	Total Accuracy
1	CHN	CHN	0.88	91.67%	94.312%
2	CHN	CHN	0.95		
3	CHN	CHN	0.99		
4	CHN	CHN	0.84		
5	CHN	CHN	0.93		
6	CHN	CHN	0.94		
7	CHN	CHN	1		
8	CHN	CHN	0.98		
9	CHN	CHN	1		
10	CHN	ARG	0.62		
11	CHN	CHN	0.82		
12	CHN	CHN	0.94		
13	ARG	ARG	0.97	100%	
14	ARG	ARG	0.99		
15	ARG	ARG	0.98		
16	ARG	ARG	0.97		
17	ARG	ARG	0.92		

**Table 5:** Prediction results of the Random Forest testing set.

Samples Number	Origin Class	Predicted Class	Confidence	Accuracy	Total Accuracy
1	CHN	CHN	0.86	100%	82.35%
2	CHN	CHN	0.9		
3	CHN	CHN	0.83		
4	CHN	CHN	0.53		
5	CHN	CHN	0.84		
6	CHN	CHN	0.86		
7	CHN	CHN	0.87		
8	CHN	CHN	0.98		
9	CHN	CHN	0.79		
10	CHN	CHN	0.72		
11	CHN	CHN	0.85		
12	CHN	CHN	0.9		
13	ARG	ARG	0.71	60%	
14	ARG	ARG	0.69		
15	ARG	ARG	0.65		
16	ARG	CHN	0.59		
17	ARG	CHN	0.58		

## Data Availability

Data will be made available on request.
